# StresSeed: The Unfolded Protein Response During Seed Development

**DOI:** 10.3389/fpls.2022.869008

**Published:** 2022-03-31

**Authors:** Alessandro Vitale, Emanuela Pedrazzini

**Affiliations:** Istituto di Biologia e Biotecnologia Agraria, Consiglio Nazionale delle Ricerche, Milano, Italy

**Keywords:** endoplasmic reticulum, molecular chaperones, protein bodies, seed storage proteins, signal transduction, unfolded protein response

## Abstract

During seed development, the endoplasmic reticulum (ER) takes care of the synthesis and structural maturation of very high amounts of storage proteins in a relatively short time. The ER must thus adjust its extension and machinery to optimize this process. The major signaling mechanism to maintain ER homeostasis is the unfolded protein response (UPR). Both storage proteins that assemble into ER-connected protein bodies and those that are delivered to protein storage vacuoles stimulate the UPR, but its extent and features are specific for the different storage protein classes and even for individual members of each class. Furthermore, evidence exists for anticipatory UPR directly connected to the development of storage seed cells and for selective degradation of certain storage proteins soon after their synthesis, whose signaling details are however still largely unknown. All these events are discussed, also in the light of known features of mammalian UPR.

## Introduction

In a relatively short time, seeds accumulate large amounts of storage proteins, destined to provide an early source of amino acids at the beginning of germination. In major crops, from around 7% (rice) up to more than 35% (soybean) of dry seed weight is constituted by proteins, mostly represented by storage proteins, encoded in each species by around a couple of dozens genes or less (Day, 2013). All storage protein polypeptides have a transient signal peptide that allows cotranslational insertion into the lumen of the endoplasmic reticulum (ER); thus, they belong to the wide class of secretory proteins, which are destined for secretion or delivery to the different compartments of the endomembrane system (Shewry et al., 1995). The cells of seed storage tissues thus rapidly become professional secretory cells, challenging the ability of the ER to take care of protein folding and assembly.

## The Unfolded Protein Response

Conditions that stress the protein folding capacity of the ER are recognized by the protein quality control system of the ER, leading to activation of the unfolded protein response (UPR), a network of signaling pathways with the main function of maintaining ER homeostasis (van Anken et al., 2021). The first UPR effects are increased expression of genes encoding ER folding helpers, of proteins implicated in traffic along the secretory pathway and in the ER-associated degradation of severely misfolded polypeptides (ERAD), as well as translational attenuation. When this fails to maintain homeostasis, UPR promotes mRNA degradation, autophagy, and finally programmed cell death. Two transmembrane UPR sensors have been characterized in the plant ER; the INOSITOL REQUIRING ENZYME1 (IRE1; Koizumi et al., 2001) and the transcription factor BASIC LEUCINE ZIPPER 28 (bZIP28; Liu et al., 2007), which are orthologs of mammalian IRE1 and ACTIVATING TRANSCRIPTION FACTOR 6 (ATF6), respectively. The luminal domains of mammalian IRE1 (Oikawa et al., 2009) and ATF6 (Shen et al., 2005) constitutively associate with the immunoglobulin binding protein (BiP), a member of the heat shock protein 70 family and the major chaperone of the ER (Liu and Howell, 2016; Pobre et al., 2019). bZIP28 also associates with BiP (Srivastava et al., 2013). To date, there is no evidence for direct interactions between plant IRE1 and BiP, but the plant sensor domain functionally replaces that of yeast IRE1, which is a BiP binder (Koizumi et al., 2001; Pincus et al., 2010). BiP recognizes short hydrophobic stretches along the primary sequence of newly synthesized polypeptides entering the ER, thus avoiding uncontrolled protein aggregation (Flynn et al., 1991). Affinity to BiP persists until the secretory polypeptide acquires proper folding and, in the case of oligomeric proteins, correct quaternary structure is formed (Bole et al., 1986; Vitale et al., 1995). There is therefore a competition for BiP binding between the UPR sensors and newly synthesized polypeptides that enter the ER: increased demands for BIP by the latter free increased amounts of sensors (Srivastava et al., 2013). IRE1 freed of BiP undergoes conformational changes that allow IRE1 dimerization, autophosphorylation, and activation of its RNAase activity (Shamu and Walter, 1996; Pincus et al., 2010). Active IRE1 performs unconventional cytoplasmic splicing on the mRNA of the bZIP60 transcription factor, which activates many UPR-responsive genes, including those of BiP and several other folding factors of the ER (Nagashima et al., 2011). IRE1 also degrades a significant proportion of mRNAs encoding secretory proteins (Mishiba et al., 2013) *via* a mechanism called regulated Ire1-dependent decay (RIDD). When BiP is released from bZIP28, this membrane-bound transcription factor traffics from the ER to the Golgi complex. Here, specific proteases release its cytosolic, active domain, which enters the nucleus and activates UPR-responsive genes (Liu et al., 2007; Iwata et al., 2017). A third BiP-binding sensor has been characterized in mammalian cells, PROTEIN KINASE RNA-LIKE ENDOPLASMIC RETICULUM KINASE (PERK; van Anken et al., 2021), which however has not been found to date in any plant. Once activated by BiP release, PERK phosphorylates the translation initiation factor eIF2α, thus attenuating general protein synthesis as a further response to ER stress. A plant alternative to this process has been identified in maize seedlings: upon ER stress part of UPR-induced mRNAs drive the formation of stress granules, which sequester other mRNAs thus transiently decreasing general translation efficiency (Kanodia et al., 2020).

An increase in BiP expression is a landmark of UPR, first observed in rapidly growing cancer cells that undergo glucose depletion, hampering cotranslational protein glycosylation in the ER (Stone et al., 1974; Welch et al., 1983; Munro and Pelham, 1986), and then found upon the synthesis of many genetically defective polypeptides or orphan subunits and when cells are treated with drugs that alter protein folding in the ER. All these conditions increase the amount of permanently misfolded or unassembled polypeptides in the ER, causing increased demand for BiP. It is however clear that BiP expression is also regulated by cell and tissue development programs. The universal paradigm of developmentally regulated UPR induction is represented by the differentiation of mammalian B cells into plasma cells as they become immunoglobulin secreting factories. Conclusive experimental evidence that such induction is the specific consequence of insufficient BiP availability has been obtained only recently, using HeLa cells engineered to produce an immunoglobulin heavy chain without its partner light chain (Vitale et al., 2019). Importantly, both in animal and plant cells it has also been directly shown that the magnitude of UPR signaling is not merely dependent on the amount of secretory protein synthesized, but to the extent to which secretory proteins sequester BiP, pointing to a fundamental role of the specific BiP avidity of a given secretory protein (Vitale et al., 2019; Brocca et al., 2021).

The following paragraph will therefore introduce the different seed storage protein classes.

## Seed Storage Protein Classes and BiP Interactions

All land plants accumulate in storage vacuoles of developing embryos two major classes of soluble storage proteins: the homooligomeric 7S/11S globulins, which form trimers in the ER, and the monomeric 2S albumins (Table 1). After folding and oligomerization in the ER, these proteins enter vesicular traffic along the secretory pathway to storage vacuoles, mainly through the Golgi complex and multivesicular bodies (Vitale and Hinz, 2005). Exceptions exists, such as those observed in pumpkin (*Cucurbita* sp), in which the storage proteins form ER-localized accretions that are then directly delivered to storage vacuoles, but also in these cases the proteins remain soluble in aqueous buffers (Hara-Nishimura et al., 1998). Vacuolar storage proteins are also present in grasses, where however they represent a minor seed protein fraction, the major and sometimes almost exclusive fraction being instead represented by prolamins (Table 1), a newly evolved storage protein class with unique biochemical and cell biology features (Xu and Messing, 2009). Prolamins have variable structures; they are divided into α, β, γ, δ, and high molecular weight (HMW) subclasses, but they have the common characteristic of forming very large insoluble heteropolymers termed protein bodies (PBs) in the ER lumen. PBs are unable to enter vesicular traffic, have a spherical shape with diameter usually between 0.5 and 2.0 microns, and retain ribosomes on their cytosolic face (Xu and Messing, 2009; Pedrazzini et al., 2016). α-globulin, a vacuolar 2S albumin-like storage protein present in all grasses, is believed to be the closest relative of prolamins (Xu and Messing, 2009), most of which are characterized by insertions/deletion or addition of new cysteine-rich domains to its structure (Pedrazzini et al., 2016). PB formation is due to hydrophobic interactions and inter-chain disulfide bonds; most individual prolamin polypeptides can be only solubilized in aqueous/alcohol or in reducing buffers, depending on the subclass (Pedrazzini et al., 2016). In Panicoideae (maize, sorghum, millet), PBs remain connected to the ER (Arcalis et al., 2020); in other cereals, such as wheat (*Triticum aestivum*), they can be delivered to protein storage vacuoles *via* autophagy-like processes (Tosi, 2012).

**Table 1:** Main features of the major classes of seed storage proteins.

Class	Biochemical features	Subcellular localization	Plants	Food plants
7S and 11S globulins	Soluble trimers and hexamers^1^	Vacuoles	Probably all seed plants and pteridophytes	Major proteins in legumes
2S albumins	Soluble monomers	Vacuoles	Probably all seed plants and pteridophytes	Major proteins in oilseed
Prolamins	Large, insoluble heteropolymers	Protein bodies mostly connected to the ER	Grasses	Major proteins in most cereals

1 *7S and 11S globulins quaternary structures are very similar. Both proteins form homotrimers in the ER. Once delivered to storage vacuoles, 11S globulin trimers assemble into hexamers*.

BiP transcripts and protein increase at early stages of soybean (*Glycine max*) embryo maturation, decreasing at late stages of seed development, thus correlating with the synthesis of soluble vacuolar storage proteins (Kalinski et al., 1995; Fontes et al., 1996). Rice (*Oryza sativa*) has five BiP genes; *BIP1*, the only one expressed to high levels in seeds, also increases its expression at the beginning of endosperm development, and BiP protein accumulation in this case persists until the end of development (Muench et al., 1997; Wakasa et al., 2011). In developing common bean (*Phaseolus vulgaris*) cotyledons, newly synthesized, unassembled polypeptides of the 7S globulin phaseolin can be co-immunorecipitated with BiP, whereas assembled trimers still present in the ER do not interact with the chaperone (Vitale et al., 1995), indicating that phaseolin assembly is assisted by BiP until trimers are formed. This was confirmed by the observation that an engineered phaseolin polypeptide unable to trimerize does not traffic from the ER and is the major BiP ligand when expressed in vegetative tissues of transgenic tobacco (*Nicotiana tabacum*; Pedrazzini et al., 1997). Similarly extended interactions had been detected between BiP and prolamins in developing rice endosperm, where BiP was found associated to the PB surface (Li et al., 1993). Thus, these studies indicated that, in general, the synthesis of storage proteins during normal seed development may challenge the protein folding capacity of the ER. Direct correlation with UPR signaling was indicated by the increase in both *bZIP60* expression and unconventional splicing when synthesis of storage proteins is active in the endosperm of maize (Gayral et al., 2017) and of *Brachypodium distachyon*, a grass evolutionarily close to wheat and barley (Kim et al., 2017).

## Tailored UPR Induction for Each Type of Storage Protein

Zeins, the prolamins of maize, belong to the α, β, γ, δ subclasses and are distributed in an ordered developmental and structural fashion in the maize PB (Lending and Larkins, 1989). γ-zeins (three polypeptides of 16, 27, and 50 kDa) and the single β-zein (18 kDa) form the outer layer of the PB, in contact with the ER membrane, and are synthesized mainly at early stages of embryo development, before the synthesis of α-zeins (more than 20 polypeptides around 19–22 kD) and of the two δ-zeins (10 and 18 kDa), which constitute the central PB core (Lending and Larkins, 1989). 27 kDa γ-zein (27γz, the most abundant zein) is soluble only upon reduction of disulfide bonds and forms homotypic, insoluble PBs also when expressed in vegetative tissues of transgenic plants (Geli et al., 1994). Its N-terminal domain contains a repeated proline-rich region and seven cysteine residues involved in inter-chain bonds; mutagenesis to serine of six of them (27γz 1C) results in full solubility also in oxidizing conditions and in traffic along the secretory pathway (Mainieri et al., 2014). The 27γz paralog 16 kD γ-zein (16γz), which is located at the interface between the outer layer and the inner PB core, originated upon the recent whole-genome duplication in maize (Xu and Messing, 2008) and underwent a large deletion in the N-terminal region, resulting in the loss of most proline-rich repeats and of three cysteine residues. When expressed ectopically in Arabidopsis (*Arabidopsis thaliana*) vegetative tissues, 16γz assembles into thread-like electron-dense structures that markedly enlarge the ER lumen, being unable to form homotypic PBs. 16γz is partially soluble also in the absence of reducing agents, but becomes fully insoluble when co-expressed with 27γz, supporting the hypothesis that 16γz establishes the contact between the outer layer and the inner core of the maize PB (Mainieri et al., 2018).

Direct comparison of the UPR-inducing properties of model prolamins and a soluble 7S globulin was recently performed by comparing the effects of 16γz, 27γz, 27γz 1C and phaseolin in vegetative tissues of transgenic Arabidopsis (Brocca et al., 2021). 16γz had the strongest effect, significantly activating IRE1-mediated splicing of the *bZIP60* transcript. Lower *bZIP60* activation, below the statistical significance level, was observed upon 27γz expression, whereas 27γz 1C and PHSL had no effects at all. The bZIP28 branch of UPR was instead not induced by any of the storage proteins, and no evidence for increased autophagy was observed. Consistent with activation of IRE1, 16γz induced transcript and protein accumulation of the three Arabidopsis BiP family members, BiP1/2 and BiP3, the ER-localized DNAJ family 3A (ERDJ3A, a BiP co-chaperone, see also below), the ER-resident HSP90 chaperone endoplasmin, and protein disulfide isomerase 1–2, all of which are folding helpers known to be induced by UPR (Brocca et al., 2021). Transcripts of *TUNICAMYCIN INDUCED PROTEIN 1 (TIN1)* were also specifically induced by 16γz; TIN1 was previously identified as the second most enhanced transcript after BIP3 upon treatment with the N-glycosylation inhibitor tunicamycin, a typical UPR-inducing chemical (Iwata et al., 2010). 27γz caused significant induction of BiP3 and ERDJ3A transcripts and of BiP1/2, endoplasmin, and ERDJ3A polypeptides, although to extents lower than those induced by 16γz. Importantly, the two traffic competent proteins 27γz 1C and PHSL had no effect on any analyzed transcript. PHSL, but not 27γz 1C, induced statistically significant accumulation of BiP1/2 and endoplasmin polypeptides, which was however much lower compared to the amounts in 16γz and also lower than that in 27γz; this may reflect *bZIP60* activation below the assay detection limit or other unknown effects. The fact that 16γz is a stronger UPR inducer than 27γz was related to the observations that it has also higher affinity to BiP and that in the absence of its partner zeins it is unable to form PBs (Mainieri et al., 2018; Brocca et al., 2021). The short sequences enriched in hydrophobic amino acids recognized by BiP are often exposed in not yet folded/assembled or misfolded polypeptides, but would normally be buried inside correctly folded and assembled proteins (Knarr et al., 1995; Foresti et al., 2003). It should be underlined that BiP-binding sequences are most probably present on the vast majority of secretory proteins: sequence frequency, individual BiP affinity, and rate of masking by polypeptide folding and assembly influence the UPR-inducing ability of each individual protein. The deletion in the N-terminal region that characterizes 16γz compared to 27γz creates sequences with higher BiP affinity (Brocca et al., 2021); this, and the inability to assemble into homotypic PBs may be the determinant for the higher UPR-inducing power of individually expressed 16γz.

A model thus emerges in which the prolamins challenge the ER folding machinery more strongly than the traffic competent vacuolar storage proteins, and this is linked to their polymerization and ER retention. The particularly strong effect of 16γz suggests that, with respect to its 27γz parent gene, this recently evolved prolamin is more dependent on partner zeins for acquiring a folded conformation that abolishes BiP binding. This is consistent with the observation that, during maturation and desiccation of transgenic Arabidopsis seeds, 16γz becomes progressively insoluble also in reducing buffers, indicating disordered aggregation (Arcalis et al., 2021).

Maize endosperm has however spare UPR inducibility, in spite of the high synthesis of prolamins. Indeed, one of the first demonstrations that challenging of the ER folding capacity induces BiP came from analysis of a number of opaque maize seed mutants. The *floury2*, defective endosperm^*^-B30, and *Mucronate* mutations lead to opaque phenotype, altered structure of maize PBs, general reduction in zein accumulation, marked increase in mRNA and protein levels of BiP and other ER folding factors in the endosperm and highly enhanced presence of BiP within the PBs (Boston et al., 1991; Marocco et al., 1991; Zhang and Boston, 1992; Hunter et al., 2002). Point mutations inactivated completely or partially, respectively, the signal peptide removal of a 22 kDa α-zein in *floury2* (Coleman et al., 1995) and of a 19 kDa α-zein in defective endosperm^*^-B30 (Kim et al., 2004). In *Mucronate*, a short deletion caused a frameshift along the sequence of 16γz that completely changed the last third of its sequence and altered its solubility (Kim et al., 2006). More recently, it was shown that in the *floury4* opaque mutant a point mutation inhibits the signal peptide removal in a 19 kDa α-zein polypeptide, induces maize *bZIP60* expression and splicing, expression of UPR-regulated folding helpers, translational suppression, and programmed cell death (Wang et al., 2014). Therefore, severe structurally defects in individual zein polypeptides induce UPR to variable extents, a further indication that heteropolymeric PB assembly is an ordered process rather than an aggregation event.

Conversely, the *opaque2* mutation, which inactivates a transcription factor that supports the synthesis of several α-zeins, leading to a substantial reduction of this zein subclass, does not have UPR effects and is epistatic on *floury2* (Galante et al., 1983; Hunter et al., 2002; Morton et al., 2016). In quality protein maize (QPM), duplication of the *27*γ*z* gene leads to much higher accumulation of its encoded zein. This restores the vitreous endosperm phenotype in *opaque2* genetic backgrounds and upregulates typical UPR-induced genes encoding ER folding helpers (Li et al., 2020), testifying the UPR induction ability of this gamma zein. *27*γ*z* duplication is an ancient event in maize evolution but it is present only in about 20% of inbred lines, indicating higher stability of the single-copy situation (Liu et al., 2019), perhaps because enhanced ER stress is a counterselected phenotype. Remarkably, when the closely linked *27*γ*z* and *50*γ*z* genes where deleted by γ-irradiation of a QPM line, two BiP mRNAs where among the twenty transcripts with at least four-fold induction (Yuan et al., 2014). The observations that both an excess or an absence of 27γz, but not the lack of most α-zein polypeptides, induce UPR in maize endosperm point to the outer layer as the fundamental structural determinant of a correctly assembled PB. This is also consistent with the earlier appearance of γ- and β-zeins compared to α-zeins during maize evolution (Xu and Messing, 2008).

Proteomic analysis of seeds of common bean mutant line SMARC1N-PN1, which lacks the major vacuolar storage proteins phaseolin, phytohemagglutinin, and arcelin, indicates that BiP level is not increased, and actually, it is decreased with a paralleled decrease of proteins involved in secretory traffic, suggesting a reduced requirement for this chaperone (Marsolais et al., 2010). Similarly to phaseolin, newly synthesized, probably not yet assembled, phytohemagglutinin is also a BiP ligand, whereas clear BiP association of arcelin and BiP is only detectable upon tunicamycin stress (Vitale et al., 1995). The absence of BiP induction in SMARC1N-PN1 parallels the data in opaque 2 in supporting a model in which mutations that inhibit the synthesis of individual proteins but do not have detrimental effects on the folding of other secretory polypeptides do not increase the requirement for BiP. However, perhaps surprisingly, proteins involved in ERAD accumulate to higher levels in SMARC1N-PN1. Even if no transcript analysis was performed, such apparent induction of an UPR process in the absence of BiP induction still needs to be elucidated. The results anyway underline that BiP levels can be modulated in relationship to the rate of the secretory flux and ER capacity.

Rice, together with oats, is an unusual cereal, in that it mainly accumulates vacuolar 11S globulins (glutelins) and α-globulin, although its PB-forming prolamins are also relatively abundant (He et al., 2021). This has facilitated comparing the cell biology of the two protein classes in a single plant. A main result was the establishment that the mRNAs of vacuolar and PB-forming storage proteins are targeted to two different ER subdomains, cisternal and PB-forming (Choi et al., 2000). Such unequal targeting is mediated by specific nucleotide sequences recognized by cytoskeleton-associated RNA-binding proteins (Tian et al., 2019; and references therein). Interestingly, also different members of the PDI family are unequally distributed in the ER of rice endosperm cells and specifically involved in the synthesis of either prolamins or vacuolar storage proteins (Onda and Kawagoe, 2011). Rice prolamins (oryzins) are produced by three gene families, encoding γ-prolamins of 13 kDa and 16 kDa, which constitute most of the PB content, and δ-prolamins of 10 kDa, which form a relatively small PB core (Xu and Messing, 2009; Saito et al., 2012). During endosperm development, OsBiP1 is enriched in PBs compared to cisternal ER, and it is specifically detected at the PB periphery by immunoelectron microscopy (Muench et al., 1997). At least five *BiP* genes are present in rice, but *BiP1* is the only one constitutively expressed at easily detectable levels in the absence of ER stress. Suppression of *BiP1* expression causes an opaque phenotype and severely reduced accumulation of major storage proteins except a 13 kDa prolamin (Wakasa et al., 2011); it also leads to induction of rice *bZIP50* (the rice ortholog of *AtbZIP60*), of several, albeit not all, folding factor genes, including *OsBiP2*, *OsBiP3,* and *OsBiP5*, and cell death genes expected to be under UPR control, thus indicating a major role of BiP1 in the synthesis of rice storage proteins and the expected role of UPR as a mechanism activated by low availability of BiP (Wakasa et al., 2011).

Members of the ER-localized DnaJ family (ERdjs) are co-chaperones that participate in BiP activity, stimulating its ATP-dependent substrate interactions and in certain cases also directly binding unfolded BiP clients (Pobre et al., 2019). Rice has six members of this family. In maturing rice endosperm, OsERdj2 follows a temporal pattern of accumulation similar to that of OsBiP1 and is detected at both the ER and the periphery of PB-I, indicating its specific involvement in PB biogenesis (Ohta et al., 2013). Conversely, OsERdj3A is localized in protein storage vacuoles, which was interpreted as suggesting its involvement in transporting misfolded proteins to vacuole, and other ERdjs are exclusively detected in the ER (Ohta et al., 2013). Antisense inhibition of *OsERdj7* expression during endosperm development induced UPR and specifically reduced the accumulation of 10 and 16 kDa oryzins as well as of α-globulin (Ohta and Takaiwa, 2020). 13 kDa oryzins and glutelins accumulated at normal levels, but two 13 kDa oryzins, RM4 and RM9, were not incorporated in the PBs and were instead mainly distributed in the ER. Together with the high induction of Arabidopsis ERdj3A in transgenic plants that express 16γz and 27γz but not in those expressing 27γz 1C or PHSL, mentioned above (Brocca et al., 2021), the picture that emerges implies that the functions of individual ERdjs in the synthesis or disposal of storage proteins is client-specific. Different ERdjs could be involved in specifically tailoring the activity of the promiscuous BIP chaperone on the individual polypeptides.

## Does UPR Keep Pace With the Synthesis of Storage Proteins?

During B cell differentiation into plasma cells, the actual start of IgM synthesis that drives UPR is preceded by an “anticipating” ER expansion and folding helpers increase (Gass et al., 2002). A similar process may occur in plants: in wheat endosperm, an increase in PDI expression precedes by several days the onset of storage protein accumulation (Shimoni et al., 1995). A relevant question is however whether this anticipatory adjustment of the ER and the following full UPR activation driven by massive storage protein synthesis are sufficient to fully support storage protein folding and accumulation.

A first indication that this may not be the case came from studies on ERAD in rice. ERAD is the first safety mechanism that UPR has to dispose of permanently misfolded newly synthesized secretory protein, the other ones being delivery to hydrolytic compartments and autophagy (van Anken et al., 2021). After many unfruitful cycles of association with ER folding factors, ERAD dislocates defective polypeptides from the ER into the cytosol and targets them to ubiquitin-mediated proteasomal degradation. In developing rice endosperm, a relevant proportion of the cysteine-rich 13 kDa prolamin RM1 was detected in a polyubiquitinated form when proteasomal activity was chemically inhibited (Ohta and Takaiwa, 2015). Importantly, RNA interference downregulation of *OsHRD3*, which is part of the ubiquitin–ligase complex involved in ERAD, caused impairment of general protein polyubiquitination, resulting in the induction of OsbZIP50 mRNA splicing and enhanced transcription of several ER folding helper genes, indicating UPR activation (Ohta and Takaiwa, 2015). Downregulation of *OsHRD3* also abolished RM1 polyubiquitination, increasing its accumulation levels. This indicates that in normal condition a proportion of RM1 polypeptides is targeted to ERAD, suggesting that they are either in excess with respect to the other PB subunits or they fail to fold properly (Ohta and Takaiwa, 2015). In line with this finding, slight *OsBiP1* overexpression in rice seeds caused increased accumulation of other chaperones and storage polypeptides, suggesting that in normal conditions the protein folding machinery is a limiting factor for the accumulation of storage proteins (Wakasa et al., 2011). However, when *OsBiP1* is highly overexpressed, the effects are very similar to those obtained with its silencing, suggesting that above a certain threshold the chaperone and sensor activities of BiP are adversely affected, possibly due to its own aggregation (Wakasa et al., 2011).

Recently, accurate measurement of the balance between protein synthesis and degradation as well as of energy costs in developing wheat grain indicated that about 25% of newly synthesized storage polypeptides was degraded, especially around 10 days after anthesis, the first stages of endosperm development (Cao et al., 2022). This is perhaps a surprising high value: given that storage protein synthesis was a major user of ATP during grain development, representing 10% of the total, it seems that a relevant amount of energy is used in an apparently futile cycle of synthesizing storage protein than will fail accumulation (Cao et al., 2022). Wheat prolamins belong to the γ (gliadins and low molecular weight glutenins) and HMW (HMW glutenins) subclasses; representatives of each class were calculated to be among the most energy-expensive wheat grain proteins in terms of both synthesis and degradation, although not all isoforms within each prolamin class were represented, and certain vacuolar storage proteins were also included (Cao et al., 2022). This indicated that certain wheat storage protein polypeptides have higher tendency to fail accumulation, similarly to what has been found in rice.

In the case of wheat, it has not been determined whether ERAD, vacuolar delivery, or traffic is involved in storage protein degradation. Interestingly, during mammalian plasma cell differentiation, autophagy is activated to degrade a proportion of the massive immunoglobulin production (Pengo et al., 2013). Genetic inhibition of autophagy leads a selective increase in the synthesis and secretion of immunoglobulins but to reduced ATP levels and increased apoptosis (Pengo et al., 2013). This indicates that the autophagic process is not activated by misfolded/aggregated polypeptides; rather, autophagy limits ER stress due to a prolonged massive antibodies production and supports ATP level by recycling immunoglobulins and other components (Pengo et al., 2013). This suggests an important role of mammalian autophagy in containing the demanding immunoglobulin synthesis in a sustainable manner, optimizing energy metabolism.

By transient transfection of rice protoplasts, it has also been demonstrated that the transcripts encoding rice 10 kDa and 16 kDa oryzalins and α-globulin are highly susceptible to RIDD upon treatment with UPR inducers (Hayashi et al., 2016). Transcripts of *glutelinA2*, as well as of UPR players, such as *BiP* and *SAR1*, are not degraded, indicating that RIDD acts on specific storage protein mRNAs and does not affect the UPR machinery. Although RIDD has normally little effect on the accumulation of storage proteins in seeds, it could intervene at the onset of ER stress to regulate their expression at post-transcriptional level.

Partially protein or mRNA degradation by professional secretory cells could therefore be a common mechanism in plants and animals to balance the best protein production efficiency with the energy requirements.

## UPR and Seed Heat Stress

Increased temperatures beyond physiological levels is perhaps the most serious abiotic insult encountered by crops. In *Brachypodium distachyon*, measurements in developing endosperm showed increases of one BiP and one PDI transcript and both spliced and unspliced bZIP60 at early developmental stages, when storage protein synthesis is particularly high, compared to other plant tissues (Kim et al., 2017). Mild heat stress (30°C) did not further increase BiP and PDI transcripts that are induced during seed development, and actually decreased those of the two chaperones (Kim et al., 2017). The authors concluded that early endosperm development specifically activates UPR but this is actually inhibited under mild heat stress, possibly because temperature increase caused a general reduction of grain filling. Indeed, in developing *Phaseolus vulgaris* cotyledons severe heat shock (43°C) induced the synthesis of cytosolic heat shock proteins as indicated by pulse-chase radioactive protein labeling, but decreased general protein synthesis, including those of BiP and endoplasmin (D’Amico et al., 1992). Heat stress (38/28°C, day/night) during grain filling generally reduced the abundance of zein transcripts; however, a marked, statistically significant reduction in zein protein accumulation was only observed for 27γz, 16γz and 19αz, with decreases of around 40, 35 and 40%, respectively (Boehlein et al., 2019). Conversely, accumulation of the other zeins analyzed was practically unaffected. This may suggest that correct folding of γ-zeins is more sensitive to heat stress and this in turn has negative effects on the 19 kDa α-zeins that directly interact with the outer PB layer mainly constituted by γ-zeins. Upon treatment of developing rice seeds with moderate heat stress (35°C) at 1 or 2 DAF, transient *OsbZIP50* transcription and splicing were enhanced within 1 h in the endosperm, with consistent increase in transcripts of folding factors, including BIP and PDI isoforms (Sandhu et al., 2021). The bZIP28 branch of UPR was instead not activated. It was therefore concluded that IRE1-mediated ER stress response participates in early heat stress response in rice seeds, although whether this is a direct result of storage protein misfolding remains to be determined. Furthermore, jasmonic acid biosynthesis and signaling genes were also induced, and treatment with the hormone induced IRE1-mediated UPR response, thus indicating relationships between UPR and jasmonic acid that however remain to be elucidated (Sandhu et al., 2021).

Overall, although heat stress is certainly an UPR inducer in vegetative tissues (Deng et al., 2013; Li et al., 2018), its relationships with UPR during seed development remain an open question.

## Challenges and Perspectives

**Figure 1:**
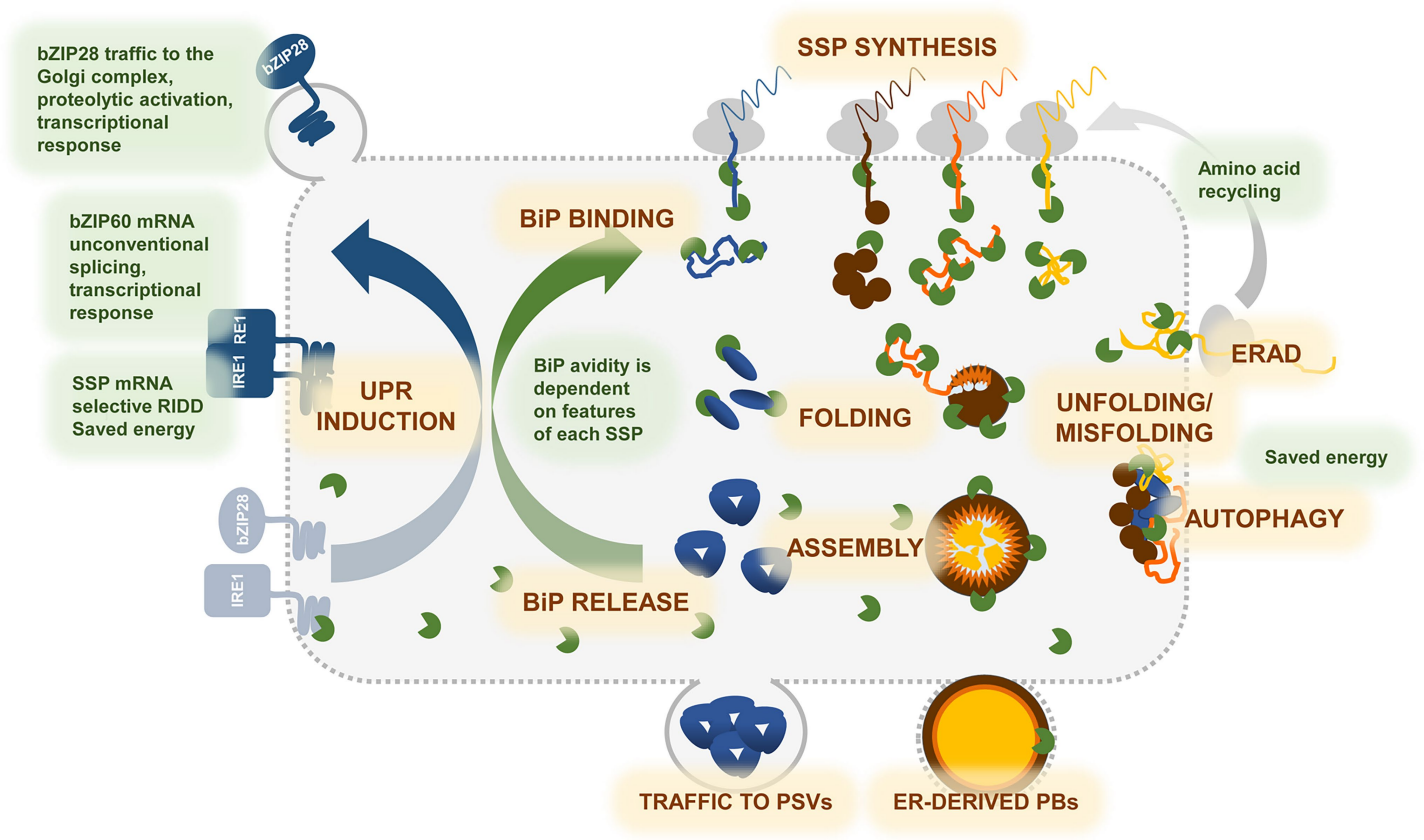
Graphic representation of the events underlying synthesis and accumulation of storage proteins in developing seeds, and cross-talk with the UPR. During seed development, massive storage proteins synthesis drives significant developmental changes in cotyledonary or endorspermal cells, transforming them in professional secretory cells. Seed storage proteins (SSPs) are cotranslationally inserted into the ER—SSP SYNTHESIS, where they undergo oxidative folding assisted by ER chaperones and enzymes—FOLDING. BiP (green) plays a major role in the folding process, masking hydrophobic regions in the unfolded SSPs, avoiding their aggregation, and guiding folding toward the correct conformation—BiP BINDING. Structure and final localization of each SSP determine its affinity for BiP and binding extent in time. Other co-chaperones, such as ERdjs, PDIs, ER lectins (not shown in the cartoon), assist and support folding in a protein-specific manner. Correct folding frees SSP monomers from BiP. In multimeric SSPs, the assembly process—ASSEMBLY—is also needed to displace BiP—BiP RELEASE. Trimeric globulins (blue polypeptides), such as phaseolin, rapidly assemble and enter traffic to the protein storage vacuoles (PSV)—TRAFFIC TO PSVs. Zein prolamins (brown, orange, and yellow polypeptides) form very large heteropolymers—ER-DERIVED PBs—that may require long time to assembly correctly and do not enter traffic. Biogenesis of prolamin PBs is therefore a process that challenges the ER machinery to a greater extent. When folding fails—UNFOLDING/MISFOLDING—SSPs which have not passed the ER quality control are degraded by ER-associated degradation—ERAD—or by AUTOPHAGY. Degradation of misfolded proteins provides amino acids to be immediately recycled into newly synthesized SSPs. Binding of BiP to the ER stress sensors IRE1 and bZIP28 links the folding process to the unfolded protein response—UPR INDUCTION. When the folding demand exceeds the ER capacity, the two sensors freed from BiP, activate the UPR pathway, that results in increasing the ER folding as well as ERAD and autophagic machineries. Selective regulated Ire1-dependent decay (RIDD) of SSP transcripts possibly also occurs to avoid ER overloading. Notice that anticipatory UPR (see main text) is not illustrated, since its signaling in seeds is not defined.

Extensive evidence is now available that the onset of storage protein synthesis induces UPR to variable levels, depending on the class of storage protein synthesized, the individual member within each class and perhaps also the tissue of accumulation, prolamins appearing in general stronger inducers than soluble vacuolar storage proteins (Figure 1). Although the evolution from vacuolar to PB storage proteins may have been favored by the fact that endosperm, but not cotyledons, is anyway a tissue destined to undergo apoptosis at the end of seed development, more severe ER stress seems the price to pay for the energy saving of accumulating storage proteins in ER-derived PBs and thus avoiding traffic to storage vacuoles. The price may be attenuated by separating ER sectors of endosperm cells devoted to PB formation from cisternal ER, the latter being therefore spared from excessive stress. Whether prolamin-induced UPR signaling is limited to the membrane surrounding PBs has however not been demonstrated. More data on the UPR-inducing properties of individual proteins are also needed, as well as a deeper biochemical analysis on the interactions between individual storage proteins, members of the BiP multigene families present in each plant species and the UPR sensors present on the ER membrane. More in general, it is not fully clear whether the expansion of members of each family of folding helper genes in plants, as opposed to animals, is just a side effect of known ancestral polyploidy of many plant genomes or reflects a fine activity and function modulation of family members.

During B cell differentiation, the anticipatory events are not directly dependent on the splicing of the mammalian bZIP60 ortholog, XBP1 (Gass et al., 2002; Ricci et al., 2021). It has been hypothesized that XBP1 could be activated by cell differentiation *per se* or to become “hypersensitive,” before its full activation due to high IgM synthesis (van Anken et al., 2021). The mammalian kinase target of rapamycin (mTOR) is an important player of anticipatory UPR during early differentiation (Benhamron et al., 2015). mTOR cross-talks with UPR but positively regulates the B cell differentiation and secretory capacity also in the absence of XBP1 (Benhamron et al., 2015). In Arabidopsis root tips, TOR activity regulates cell differentiation and growth of root tip cells and functionally interacts with IRE1 (Angelos and Brandizzi, 2022); however, in this case, IRE1 limits uncontrolled activity of TOR. A possible role of TOR in differentiating cells of seed storage tissues, in promoting ER expansion and increasing the abundance of folding helpers that may precede storage protein synthesis remains to be demonstrated.

Two apparently contradicting aspects are also emerging. On one side, the analysis of many mutants has clearly indicated that the UPR capacity is not saturated by storage protein synthesis, suggesting that normally the folding capacity of the ER keeps pace with the elevated levels of synthesis. On the other side, evidence is growing that a not irrelevant proportion of newly synthesized storage proteins are degraded during seed development, strongly suggesting failure to fold and assemble properly and, more importantly, that this situation does not stimulate UPR to its maximal extent (Figure 1). More research is needed on determining the specific features of individual polypeptides that are most subjected to degradation. It should also be verified whether such high extent of degradation has positive energy functions, as suggested in the case of mammalian plasma cells (Pengo et al., 2013), or has other unknown signaling functions. Regarding the first hypothesis, it should be taken into account that in developing wheat grain total protein degradation is not *per se* highly energy consuming, since it only uses one-tenth of the ATP budget employed in protein synthesis (Cao et al., 2022).

The phaseolin and lectin storage proteins of Lima bean (*Phaseolus lunatus*) share a high degree of homology with those of common bean, but they are more extensively N-glycosylated and abundant. Tunicamycin treatment of common bean cotyledons does not affect traffic of the unglycosylated storage proteins to storage vacuoles (Vitale et al., 1984; D’Amico et al., 1992), but similar treatment of lima bean cotyledons leads to accumulation of phaseolin and lectins as aggregates in an enlarged ER, in which BiP remained trapped, whereas traffic of the 11S storage globulin legumin, which does not contain N-glycosylation sites, is unaffected (Sparvoli et al., 2000). Also, suppression of the vacuolar 7S storage globulin subunits of soybean alters vacuolar traffic and accumulation of other storage proteins, leading to the formation of ER accretions that morphologically resemble PBs and contain the 11S storage globulin as major component (Kinney et al., 2001). Altogether, these results underline the delicate equilibrium of storage protein folding, highly variable even between closely related polypeptides of vacuolar storage proteins, as we discussed for prolamins.

Whether the protein folding machinery and UPR sensors can be manipulated with the aim of improving seed protein quality and quantity constitutes an important challenge, also in light of the use of seeds for the production of recombinant pharmaceuticals (Arcalis et al., 2014; Panting et al., 2021). As it has been underlined (Cao et al., 2022), the loss of storage protein soon after synthesis opens perspectives for possible crop productivity improvement by conventional breeding or genetic engineering. The substitution of highly degraded polypeptides with others that are less prone to immediate degradation could in theory improve protein content per seed. An additional strategy is suggested by the recent finding that natural Arabidopsis accessions of different geographical origin exhibit different levels of IRE1-mediated UPR upon abiotic and biotic stress (Afrin et al., 2020), and that most tropical or subtropical maize inbred lines more proficiently upregulate the expression of *ZmbZIP60* in response to heat stress compared to lines used in temperate climates (Li et al., 2018). In both cases, measurements were made during vegetative growth, but these results indicate intraspecies UPR biodiversity that could also affect storage protein synthesis and accumulation.

## Author Contributions

All authors listed have made a substantial, direct, and intellectual contribution to the work and approved it for publication.

## Funding

Supported by the projects CNR-DISBA Cambiamenti Climatici 7009-FOE 2019 and PORFESR 2014-2020 Reg. Lombardia sPATIALS3 1 176485.

## Conflict of Interest

The authors declare that the research was conducted in the absence of any commercial or financial relationships that could be construed as a potential conflict of interest.

## Publisher’s Note

All claims expressed in this article are solely those of the authors and do not necessarily represent those of their affiliated organizations, or those of the publisher, the editors and the reviewers. Any product that may be evaluated in this article, or claim that may be made by its manufacturer, is not guaranteed or endorsed by the publisher.
